# Effect of Tris, MOPS, and phosphate buffers on the hydrolysis of polyethylene terephthalate films by polyester hydrolases

**DOI:** 10.1002/2211-5463.12097

**Published:** 2016-07-20

**Authors:** Juliane Schmidt, Ren Wei, Thorsten Oeser, Matheus Regis Belisário‐Ferrari, Markus Barth, Johannes Then, Wolfgang Zimmermann

**Affiliations:** ^1^ Department of Microbiology and Bioprocess TechnologyInstitute of BiochemistryLeipzig UniversityGermany

**Keywords:** 3‐(*N*‐morpholino)propanesulfonic acid, biocatalysis, inhibition, polyester hydrolase, polyethylene terephthalate, Tris

## Abstract

The enzymatic degradation of polyethylene terephthalate (PET) occurs at mild reaction conditions and may find applications in environmentally friendly plastic waste recycling processes. The hydrolytic activity of the homologous polyester hydrolases LC cutinase (LCC) from a compost metagenome and TfCut2 from *Thermobifida fusca* KW3 against PET films was strongly influenced by the reaction medium buffers tris(hydroxymethyl)aminomethane (Tris), 3‐(*N*‐morpholino)propanesulfonic acid (MOPS), and sodium phosphate. LCC showed the highest initial hydrolysis rate of PET films in 0.2 m Tris, while the rate of TfCut2 was 2.1‐fold lower at this buffer concentration. At a Tris concentration of 1 m, the hydrolysis rate of LCC decreased by more than 90% and of TfCut2 by about 80%. In 0.2 m MOPS or sodium phosphate buffer, no significant differences in the maximum initial hydrolysis rates of PET films by both enzymes were detected. When the concentration of MOPS was increased to 1 m, the hydrolysis rate of LCC decreased by about 90%. The activity of TfCut2 remained low compared to the increasing hydrolysis rates observed at higher concentrations of sodium phosphate buffer. In contrast, the activity of LCC did not change at different concentrations of this buffer. An inhibition study suggested a competitive inhibition of TfCut2 and LCC by Tris and MOPS. Molecular docking showed that Tris and MOPS interfered with the binding of the polymeric substrate in a groove located at the protein surface. A comparison of the *K*_i_ values and the average binding energies indicated MOPS as the stronger inhibitor of the both enzymes.

**Abbreviations**
BHET: bis‐(2‐hydroxyethyl) terephthalate
LCC: LC cutinase
MHET: mono‐(2‐hydroxyethyl) terephthalate
MOPS: 3‐(*N*‐morpholino)propanesulfonic acid
PET: polyethylene terephthalate
TPA: terephthalic acid

Polyethylene terephthalate is a synthetic aromatic polyester composed of ethylene glycol and terephthalic acid (TPA) 1. Because of its versatile properties PET is used in many products such as textile fibers or beverage bottles. The high strength, low weight, low permeability of gases, and its resistance to many chemicals make PET an excellent packaging material for a wide range of products 2. In 2013, about 13% of all packaging materials were made of PET 3. The widespread use of synthetic polyesters such as PET contributes to growing amounts of postconsumer plastic wastes. The related environmental pollution problems require efficient recycling processes. The biocatalytic degradation of PET offers a less energy‐consuming and environmentally friendly method than conventional recycling processes 4, 5. Hydrolytic activity against PET has been shown for lipases 1, 5, 6, 7, carboxylesterases 6, 7, 8, 9, and cutinases 1, 6, 7, 10. Several polyester hydrolases from fungi 1, actinomycetes 5, 11, 12, 13, 14, 15, 16, 17, 18, 19, and from a compost metagenome 10, 20 have been reported.

The type and ionic strength of buffers can influence the enzymatic activity in aqueous reaction systems. The effect of ions and inorganic salts on the solubility of proteins has been reported more than 120 years ago, when Hofmeister proposed a series of cations and anions according to their ability to precipitate hen egg white 21. It has been shown that Hofmeister effects can also influence the catalytic activity of enzymes. The ionic strength as well as the pH of a buffer system affect the activity and structural features of enzymes. The activity of immobilized *Candida rugosa* and *Rhizopus oryzae* lipases was influenced by the ionic strength of the buffer and was highest in a mixture of 0.25 m MOPS and sodium phosphate 22. In another study, both buffers as well as salt ions showed specific Hofmeister effects on the enzymatic activity of the *C. rugosa* lipase 23. Weak and strong electrolytes strongly influenced the enzymatic activity of the lipase. While sulfate ions increased the activity, chloride behaved neutrally and thiocyanate strongly decreased it. The influence of inorganic salts and ions on the stability and activity of alkaline phosphatase from calf intestine has also been shown 24. The activating and stabilizing effect of different inorganic salts correlated well with their kosmotropic and chaotropic properties proposed in the Hofmeister series. An influence of the ionic strength of the buffer system on the hydrolysis of PET films in a membrane reactor by the polyester hydrolase TfCut2 has also been reported 25. The highest initial hydrolysis rates were obtained in Na_2_HPO_4_ buffer at a concentration of 0.7 m.

An inhibition of the activity of aminopeptidases and aminotransferases 26, 27, 28, cholinesterases 29, and different carbohydrate hydrolases 30, 31, 32, 33, 34, 35 by Tris has been reported. Most of these studies indicated a competitive inhibition of the enzymes by this buffer. MOPS has been shown to inhibit the activity of bovine adrenal tyrosine hydroxylase 36. The related sulfonic acid buffer 4‐morpholinoethanesulfonic acid (MES) also inhibited the metallo‐β‐lactamase from *Bacteroides fragilis* 37.

Previously, we have examined the effect of the ionic strength of HEPES, MOPS, PIPES, Tris, and sodium phosphate buffers on the enzymatic hydrolysis of PET films in a membrane reactor 25. In this study, we investigate the effect of these buffer types on the hydrolysis of PET by two polyester hydrolases in further detail. The degradation of amorphous PET films by TfCut2 and LCC at different buffer concentrations was monitored by measuring the released products by RP‐HPLC. An inhibitory effect of Tris and MOPS buffer on the two hydrolases was investigated experimentally as well as by docking experiments.

## Materials and methods

### Genes, enzymes and chemicals

The synthetic LCC gene construct with adapted codon usage for *Escherichia coli* was obtained from GeneArt Gene Synthesis (Life Technologies GmbH, Darmstadt, Germany). Amorphous PET films (250 μm thickness) were purchased from Goodfellow GmbH (Bad Nauheim, Germany, product number 029‐198‐54). FastDigest restriction enzymes were purchased from Life Technologies GmbH. All other chemicals were obtained from Carl Roth GmbH + Co. KG (Karlsruhe, Germany) and Gruessing GmbH Analytica (Filsum, Germany) at the highest purity available.

### Cloning and expression of the polyester hydrolase genes

The synthetic LCC gene construct without the secretion signal peptide (ENA: LN879395) was amplified using the primers LCC‐FW (5′‐TTTTGGATCCGTCTAACCCGTACCAGCGTG‐3′) and LCC‐RV (5′‐TTTTGAATTCCCCTGGCAGTGACGGTTGTT G‐3′). The gene for TfCut2 (ENA: FR727681) without signal peptide was amplified from *T. fusca* KW3 genomic DNA using the primers TfCut2‐FW (5′‐TTTTTTGGATCCGGCCAACCCCTACGAGCGC‐3′) and TfCut2‐RV (5′‐TTTTTGAATTCGGGTAGAACGGGCAGGTGG AGC‐3′) (the restriction sites for *Bam*HI or *Eco*RI are underlined for each primer). The amplified genes were digested with FastDigest *Bam*HI and *Eco*RI and ligated into the *Bam*HI and *Eco*RI restriction sites of the vector pET‐20b(+). The final pET‐20b(+) construct containing the ligated genes, pelB leader sequence, and His_6_ tag was cloned into *E. coli* BL21 (DE3). The expression and purification of the recombinant hydrolases was carried out as described previously 38. After purification, the enzymes were concentrated and the buffer was changed to 10 mm Tris–HCl (pH 8.0 at 60 °C) using Amicon Ultra Centrifugal Filter Units (Merck KGaA, Darmstadt, Germany).

### Hydrolysis of PET films by LCC and TfCut2

Polyethylene terephthalate films of 9 cm^2^ (about 150 mg) were added to reaction vials containing 0.1–2.8 μg·cm^−2^ of purified LCC or TfCut2 and 0.1–1 m Tris, sodium phosphate or MOPS buffer (pH 8.0) in a total volume of 1.8 mL. The pH of the buffers was adjusted at 60 °C using HCl for Tris and NaOH for MOPS buffer. The reaction vials were incubated at 60 °C on a thermo shaker (1000 rpm) for 1 h. Released hydrolysis products were quantified by RP‐HPLC 39. The sum of the released soluble products—TPA, mono‐(2‐hydroxyethyl) terephthalate (MHET), and bis‐(2‐hydroxyethyl) terephthalate (BHET) was used to determine the initial hydrolysis rate. All initial rates were determined at least in triplicate.

### Inhibition of LCC and TfCut2 by Tris and MOPS

Polyethylene terephthalate films of 1–9 cm^2^ (about 20–150 mg) were added to reaction vials containing purified LCC (1 μg) or TfCut2 (5 μg) and 0.2 m sodium phosphate buffer (pH 8.0) in a total volume of 1.8 mL. Tris (0.2–0.4 m, pH 8.0) and MOPS (0.05–0.3 m, pH 8.0) were added to the reaction mixture. The vials were incubated at 60 °C on a thermo shaker (1000 rpm) for 1 h. Released hydrolysis products were quantified by RP‐HPLC 39.

### Molecular docking of Tris and MOPS to LCC and TfCut2

The crystal structures of TfCut2 (PDB: 4CG1) 40 and LCC (PDB: 4EB0) 20 were used for the docking experiments with AutoDock Vina 41. The 3D conformer structures of neutral Tris (CID 6503) 42 and protonated MOPS (CID 2723950) 43 were obtained from the Open Chemistry Database 44. The ligand structure of a PET model substrate (2PET) consisting of two units of the monomer ethylene terephthalate was built with MOE (Chemical Computing Group, Montreal, Canada). AutoDockTools (v1.5.6) (Molecular Graphics Laboratory, Department of Molecular Biology, The Scripps Research Institute, La Jolla, CA, USA) was used to prepare the files and select the search space. PyMOL 1.1r1 45 was used for the visualization of the results. The main binding sites were selected and the average binding energy was calculated.

## Results

### Effect of enzyme concentration and buffer composition on the hydrolysis of PET films by TfCut2 and LCC

**Figure 1:**
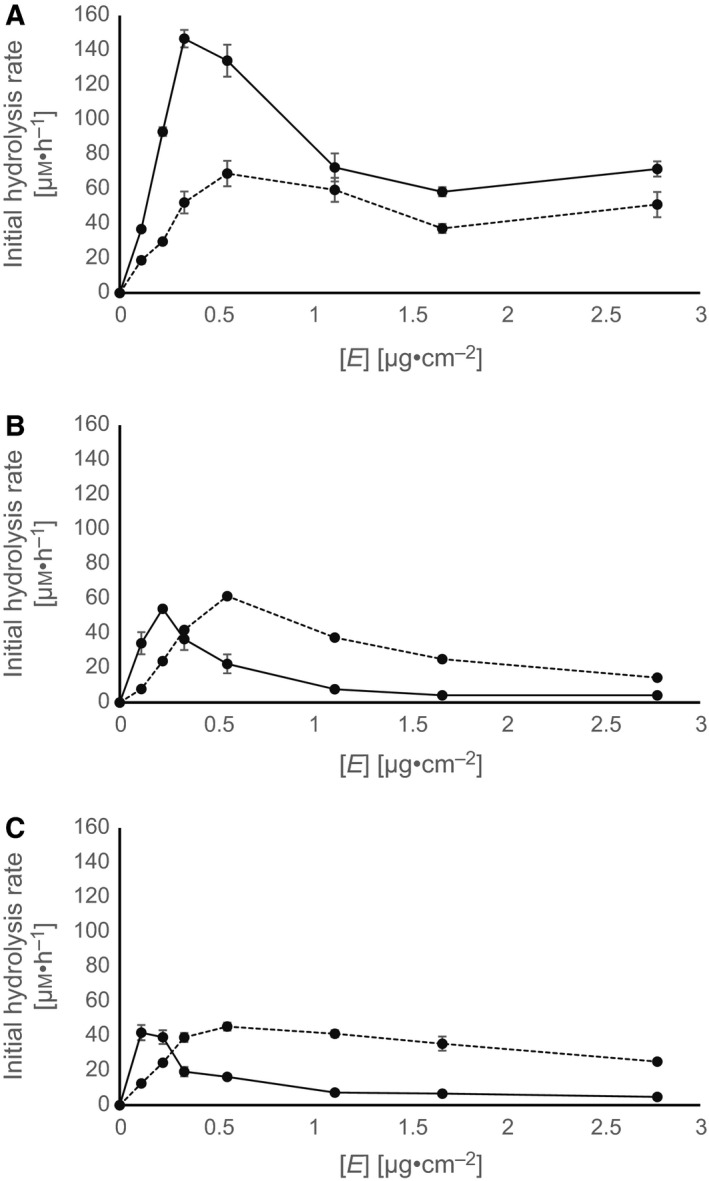
Initial hydrolysis rates of PET films (9 cm^2^) as a function of the concentration of TfCut2 (dashed line) and LCC (solid line) in (A) Tris; (B) MOPS; (C) sodium phosphate (0.2 m, pH 8.0). Error bars indicate the standard deviation of triplicate determinations.

When the hydrolysis of PET films was performed at different concentrations of TfCut2 and LCC in Tris–HCl, MOPS, and sodium phosphate buffers (0.2 m, pH 8.0), the initial hydrolysis rates increased with increasing enzyme concentrations until a maximum was reached and then leveled off or decreased at higher enzyme concentrations (Fig. 1). The maximum initial hydrolysis rates were reached with lower LCC concentrations (0.1–0.3 μg·cm^−2^) compared to TfCut2 (0.6 μg·cm^−2^) in all three buffers. LCC showed the highest activity in Tris–HCl buffer (146.6 μm·h^−1^) with a 2.1‐fold higher rate than TfCut2 (Fig. 1A). In 0.2 m MOPS and sodium phosphate, both enzymes did not significantly differ in their maximum hydrolysis rates (40–60 μm·h^−1^) (Fig. 1B,C).

### Effect of buffer concentrations on the hydrolysis of PET films by TfCut2 and LCC

**Figure 2:**
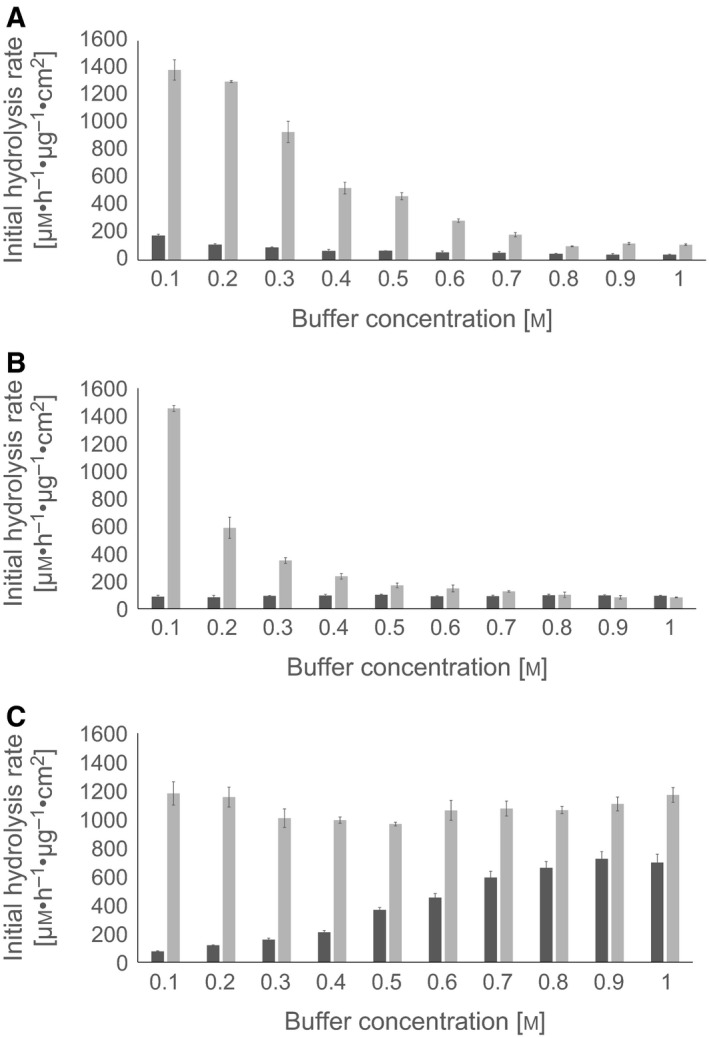
Initial hydrolysis rates of PET films (9 cm^2^) of LCC (light bars) and TfCut2 (dark bars) as a function of buffer concentration of (A) Tris, (B) MOPS, and (C) sodium phosphate (pH 8.0). In each buffer, LCC and TfCut2 were employed in concentrations corresponding to their maximum initial hydrolysis rates (see Fig. 1). Error bars indicate the standard deviation of triplicate determinations.

The effect of the buffer concentration on the hydrolysis rate of LCC and TfCut2 against PET films is shown in Fig. 2. LCC and TfCut2 were employed in concentrations corresponding to their maximum initial hydrolysis rates (see Fig. 1). The activity of TfCut2 and LCC depended on the concentration of the buffers. The activity of TfCut2 in MOPS buffer was low in the range from 0.1 to 1 m. In 1 m Tris buffer, its activity decreased by 80% compared to 0.1 m Tris buffer. In contrast, the hydrolysis rate was about 10‐fold higher at 1 m sodium phosphate compared to 0.1 m. LCC showed a different response to changing buffer concentrations. While the hydrolysis rates were unaffected within 0.1–1 m of the phosphate buffer, in 1 m Tris and MOPS buffers its activity decreased by more than 90% compared to 0.1 m.

### Inhibition of the hydrolysis of PET by LCC and TfCut2 in the presence of Tris and MOPS

The low hydrolysis rates detected with Tris and MOPS buffers suggested an inhibitory effect on the hydrolysis of PET by LCC and TfCut2. The inhibitory effect of the buffers was investigated by performing the hydrolysis reaction in 0.2 m sodium phosphate buffer (pH 8.0) containing 0.2 and 0.4 m of Tris for both enzymes or 0.1 and 0.3 m of MOPS for LCC. Since the hydrolysis rates of PET by TfCut2 at MOPS concentrations of 0.1–1 m were very low, buffer concentrations in the range from 0.05–0.075 m were used. LCC and TfCut2 were employed in concentrations corresponding to their maximum initial hydrolysis rates (see Fig. 1).

**Figure 3:**
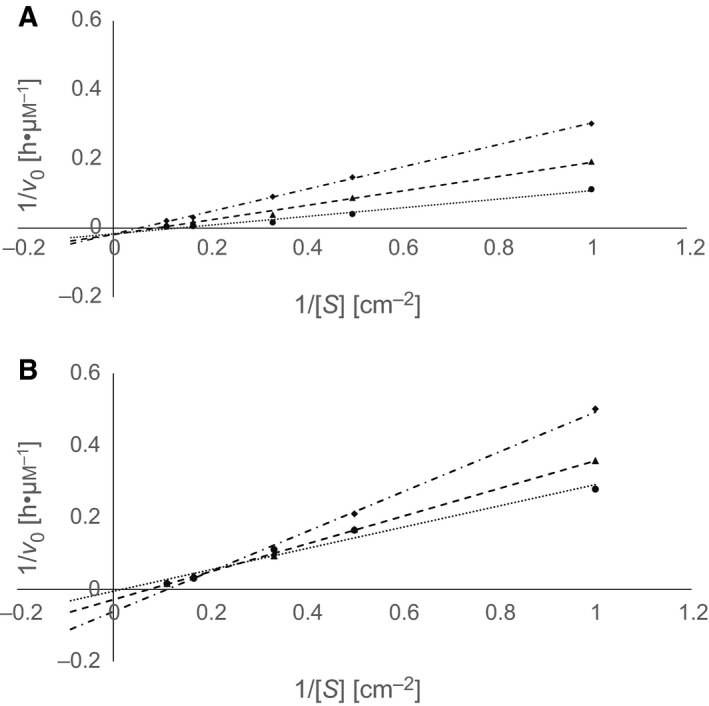
Double reciprocal plots of initial hydrolysis rates of PET films versus substrate concentration for LCC (A) and TfCut2 (B) at different concentrations of Tris: ● 0 m, ▲ 0.2 m, and ♦ 0.4 m.

**Figure 4:**
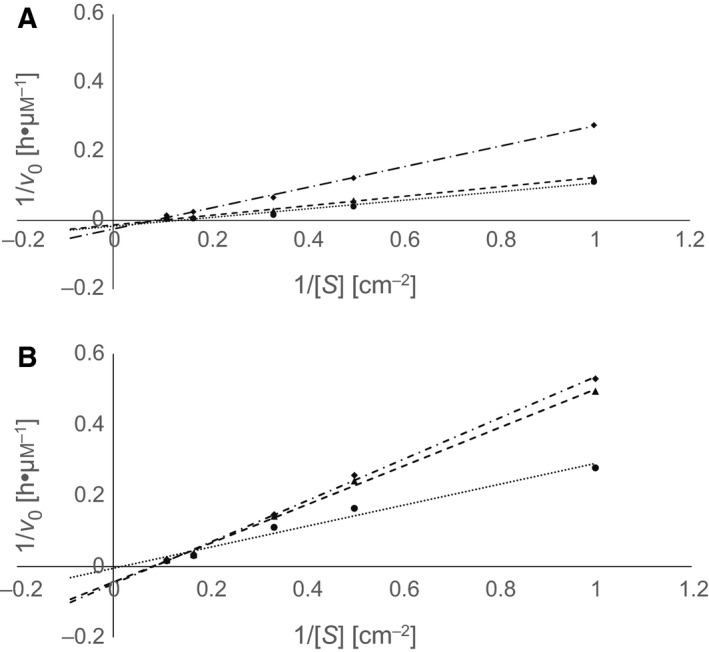
Double reciprocal plots of initial hydrolysis rates of PET films versus substrate concentration for LCC (A) and TfCut2 (B) at different concentrations of MOPS: (A) ● 0 m, ▲ 0.2 m, and ♦ 0.4 m and (B) ● 0 m, ▲ 0.05 m, and ♦ 0.075 m.

The Lineweaver–Burk plots indicated an inhibition of both enzymes by Tris and MOPS (Figs 3 and 4). All curves showed common intercepts located mainly in the first square of the plots suggesting a competitive type of inhibition of LCC and TfCut2 by Tris and MOPS. A similar result has been reported previously indicating a competitive inhibition of TfCut2 by the PET hydrolysis products MHET and BHET 39.

The corresponding *K*_i_ values (Table 1) for both buffers were determined by replotting the slopes calculated from the Lineweaver–Burk plots against the concentration of Tris and MOPS 46, 47. The results showed that MOPS is the stronger inhibitor of both enzymes with significantly lower *K*_i_ values compared to Tris.

**Table 1:** Inhibition of the hydrolysis of PET films by LCC and TfCut2 through Tris and MOPS. The *K*_i_ values were determined by replotting the slopes calculated from the Lineweaver–Burk plots against the concentration of Tris and MOPS

Inhibitor	Enzyme	*K*_i_ [m]
Tris	LCC	0.24
	TfCut2	0.44
MOPS	LCC	0.17
	TfCut2	0.08

### Molecular docking of Tris and MOPS

**Figure 5:**
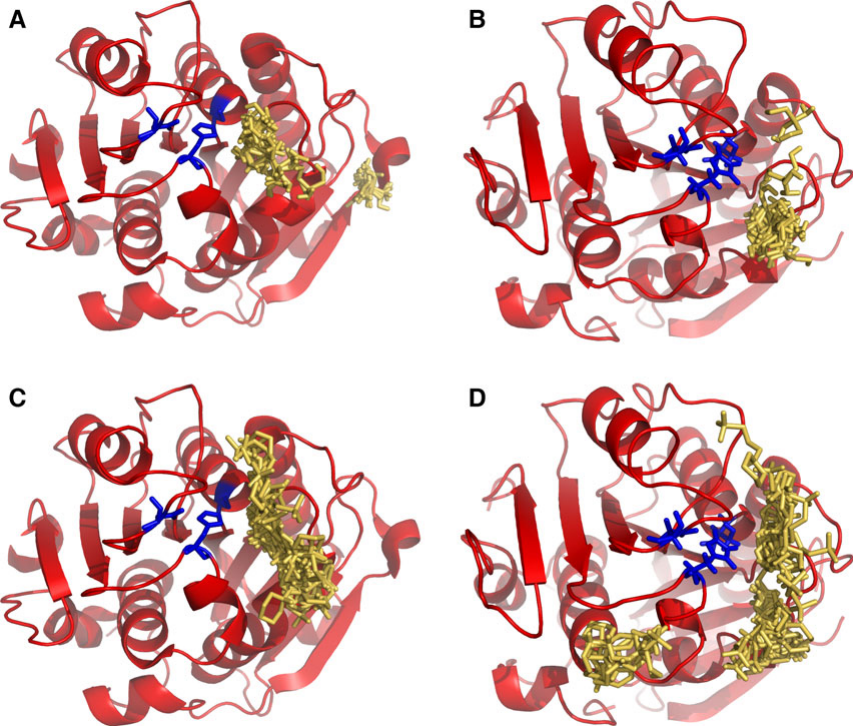
Docking of Tris and MOPS to LCC and TfCut2. An overlay of multiple binding modes of the inhibitors is presented. The structures of the enzymes are shown in red with the catalytic triad highlighted as blue sticks and Tris and MOPS as yellow sticks. (A) docking of Tris (neutral) to LCC; (B) docking of Tris (neutral) to TfCut2; (C) docking of MOPS to LCC; (D) docking of MOPS to TfCut2. The docking was performed with AutoDock Vina 41.

The molecular docking experiments performed for LCC and TfCut2 with the two inhibitors, Tris and MOPS, revealed several binding sites on the surface of the two enzymes. An interaction of the inhibitors with specific amino acid residues could not be confirmed (Fig. 5). The main binding site of Tris and MOPS was detected near the catalytic triad inside the substrate‐binding groove at the surface of LCC and TfCut2 48. A second minor binding site of Tris was located next to the substrate‐binding pocket of LCC (Fig. 5A). This site is assumed to be inside an extended region of the substrate‐binding pocket of LCC when compared to the substrate‐binding regions of the polyester hydrolases from *Thermobifida cellulosilytica* DSM44535 16. A similar minor binding site of MOPS was observed in TfCut2 (Fig. 5D). The docking experiments with Tris were carried out in its protonated and neutral state since both are present at pH 8.0. The same binding areas were obtained for both states and only the results of neutral Tris are shown in Fig. 5.

A determination of the average binding energies showed for MOPS −3.7 kcal·mol^−1^ bound to LCC and −3.5 kcal·mol^−1^ bound to TfCut2. For Tris, −3.1 kcal·mol^−1^ were obtained bound to LCC and −2.8 kcal·mol^−1^ bound to TfCut2. The average binding energies indicated a higher affinity of MOPS for both enzymes and confirmed the lower *K*_i_ values of MOPS (Table 1).

## Discussion

In this study, the effect of MOPS, Tris and sodium phosphate buffers on the enzymatic activity of the polyester hydrolases LCC and TfCut2 was investigated. The determination of the initial hydrolysis rates showed that maximum values were obtained at lower concentrations of LCC than of TfCut2 indicating a higher activity of LCC against the PET film. In 0.2 m Tris LCC showed a 2.1‐fold higher maximum initial hydrolysis rate than TfCut2. In contrast, both enzymes revealed no significant difference in their maximum hydrolysis rates in 0.2 m MOPS or sodium phosphate buffer. At increasing concentrations of Tris and MOPS buffer, the initial PET hydrolysis rates decreased or were constantly low for LCC and TfCut2. An inverse effect was observed with sodium phosphate buffer. TfCut2 showed an increased initial hydrolysis rate with increasing sodium phosphate concentrations while LCC showed constantly high initial hydrolysis rates at all tested concentrations. The kosmotropic phosphate anions possibly stabilized LCC and TfCut2 resulting in higher hydrolysis rates of the PET films. The precipitating (salting out) and solubilizing (salting in) properties of anions have been explained by different water adsorbing effects of salts 21. Ions are thereby classified according to their ability to form or break water structures. Kosmotropic (precipitating) ions stabilize proteins and support the formation of polar water structures, whereas chaotropic (solubilizing) ions break the water structures and destabilize proteins 49, 50, 51, 52. Such a stabilizing effect has been reported previously for a tetrameric maize leaf phosphoenolpyruvate carboyxlase 53. The authors suggested that kosmotropic anions such as $\text{HP}O_{4}^{2-}$ stabilized the enzyme most effectively by their water‐structuring effects and by increasing the surface tension. The observed stabilizing effect also depended on the concentration of the kosmotropic salts. $\text{HP}O_{4}^{2-}$ significantly improved the stability and activity of an endoxylanase from *Bacillus sp*. 54. By increasing the concentration of K_2_HPO_4_, an increased *T*_*m*_ value and an increase in xylanase activity was observed. The authors suggested that the activity increase and the stabilization of the enzyme by K_2_HPO_4_ was due to a conformational change caused by the phosphate anion. Supporting these findings, an activating effect of sodium phosphate buffer was also observed for LCC and TfCut2 (Fig. 2C).

While TfCut2 showed the highest initial hydrolysis rate in sodium phosphate buffer at a concentration of > 0.7 m, the hydrolysis rate of LCC was independent from the concentration of the sodium phosphate buffer in a range from 0.1 to 1 m. Since TPA is released during the hydrolysis of PET, a buffer of high molarity or strength is required to maintain the pH of the reaction medium 55. The ionic strength of the buffer has been shown previously to influence the hydrolysis of PET films by TfCut2 in an ultrafiltration membrane reactor 25. The enzyme showed a 3.8‐fold higher initial hydrolysis rate in 0.5 m Na_2_HPO_4_ than in 0.5 m Tris buffer. Confirming our results, the highest hydrolysis rate was also obtained at a sodium phosphate concentration of 0.7 m. The effect of the Na_2_HPO_4_ buffer on the hydrolytic activity of TfCut2 was attributed to the high ionic strength of the buffer 25. At 0.5 m, the Na_2_HPO_4_ buffer has an ionic strength of 3 m, while the ionic strength of Tris buffer with this concentration is 0.07 m. When the ionic strength of Tris buffer was increased to 2 m, a 2.4‐fold higher hydrolysis rate of TfCut2 was observed.

In contrast to sodium phosphate buffer, higher concentrations of Tris and MOPS resulted in a reduction of the hydrolytic activity of LCC and TfCu2 against PET suggesting an inhibitory effect of the buffers (Fig. 2A,B). The double reciprocal plots suggested a competitive inhibition. A comparison of the *K*_i_ values indicated MOPS as a stronger inhibitor for both enzymes (Table 1). When comparing the initial hydrolysis rates of PET films by TfCut2 and LCC in 0.1–1 m Tris and MOPS buffers, MOPS also caused a decrease in the hydrolysis rates at lower concentrations than Tris (Fig. 2). An inhibition of cholinesterases by Tris has been already reported in the 1960s 29. The authors showed a competitive inhibition by Tris with inhibition constants of 13–14 mm. A competitive inhibition of an α‐amylase from *Bacillus licheniformis* by Tris with a *K*_i_ of 13.12 mm has also been reported 46. Accompanying docking studies indicated a high binding potential of Tris at the active site of the enzyme. The catalytic residues Asp 174 and Glu 200 of a psychrophilic α‐amylase from *Alteromonas haloplanctis* also showed a strong interaction with the amino group of Tris 56. In addition, the hydroxyl groups of Tris were found to form hydrogen bonds with the three catalytic amino acids of the enzyme. The *K*_i_ values obtained in this study for the inhibition of LCC and TfCut2 by Tris (Table 1) are higher than those described in other reports before, suggesting a weaker inhibiting effect than for other enzymes.

Only few reports are available about an inhibitory effect of MOPS on enzyme activity. MOPS has been shown to reduce the activity of bovine adrenal tyrosine hydroxylase by 40% 36. A metallo‐β‐lactamase from *Bacteroides fragilis* was inhibited by MES which is similar to MOPS a sulfonic acid buffer 37. The crystal structure of a complex of the enzyme with MES suggested an interaction of MES with the active site of the enzyme and a competitive inhibition by MES with a *K*_i_ of 23 ± 5 mm was observed.

The molecular docking experiments performed in this study indicated that both Tris and MOPS did not bind specifically to TfCut2 and LCC. The main binding sites of Tris and MOPS were located near the catalytic triad inside the substrate‐binding groove at the surface of the two enzymes. A second minor binding site of Tris inside an extended region of the substrate‐binding pocket was present in LCC. TfCut2 showed a similar second minor binding area for MOPS. Both areas were located in a long groove at the surface of the enzymes. This groove has been proposed to play a crucial role in the recognition and accommodation of polymeric substrates by the highly homologous polyester hydrolase Est119 from *Thermobibifida alba* AHK119 48. The interaction of Tris and MOPS with this groove in LCC and TfCut2 could prevent the binding of the polymeric PET substrate resulting in an inhibition of their hydrolytic activity against PET in an apparently competitive manner. A specific interaction with amino acids of the groove could, however, not be detected, confirming the weak inhibition indicated by high *K*_i_ values for Tris and MOPS. With both enzymes, the average binding energy was 2‐ and 0.5‐fold lower for Tris and MOPS, respectively, compared to the average binding energy for the PET model compound 2PET 40, suggesting a more favorable interaction with the model substrate (data not shown).

In conclusion, the activity of the polyester hydrolases LCC and TfCut2 against PET films was shown to strongly depend on the type and concentration of the buffer. A buffer of high molarity and strength was required to stabilize the pH during the reaction. High initial hydrolysis rates were obtained using sodium phosphate buffer at concentrations > 0.7 m. In contrast, the hydrolytic activity of both enzymes was inhibited at higher concentrations of MOPS and Tris.

## Author contributions

JS, RW, TO, and WZ wrote the paper and conceived and designed the project. JS, MB, MRBF, and JT acquired and interpreted the data.
